# Estimation of mass and radii for charged compact objects using a modified Chaplygin equation of state in the Buchdahl-I metric

**DOI:** 10.1371/journal.pone.0321111

**Published:** 2025-05-20

**Authors:** A. Zahra, S. A. Mardan, Sana Saleem, Muhammad Bilal Riaz, Tomas Kozubek

**Affiliations:** 1 IT4Innovations, VSB-Technical University of Ostrava, Ostrava, Czech Republic; 2 Department of Mathematics, University of the Management and Technology, C-II, Lahore, Pakistan; 3 Chitkara University Institute of Engineering and Technology, Chitkara University, Rajpura, Punjab, India; 4 Jadara University Research Center, Jadara University, Jordan; UNITED STATES OF AMERICA

## Abstract

In this article, a class of static configurations for stellar equilibrium in relativistic charged spheres with anisotropic fluid is studied. The Buchdahl ansatz is employed to solve the Einstein-Maxwell field equations, which govern the behavior of charged, relativistic stellar objects. The matter distribution within the charged sphere is shown to satisfy all the necessary energy conditions, including the hydrostatic equilibrium condition. Several compact objects, such as GW 190814, PSR J0952-0607, PSR J0030+0451, PSR J0740+6620, GW 170817, PSR J1614-2230, PSR J2215+5135, and 4U 1608-52, are discussed to predict their masses and radii. These predictions are crucial for understanding the properties of compact stars, including neutron stars and possibly exotic stars. The physical properties of the charged sphere are examined, including mass, surface redshift, adiabatic index, and the speed of sound. The solutions are presented graphically, illustrating the structure of the stars. The results demonstrate that the maximum density and pressure occur at the center of the star, and these quantities are continuous and well-behaved throughout the star’s interior, avoiding singularities. These features offer strong support for the physical viability of the model, suggesting that the Buchdahl ansatz provides a realistic description of compact stars with electric charge and anisotropy.

## Introduction

The Einstein-Maxwell field equations (EMFEs) play an essential role in studying the interaction of gravity and electromagnetism within the context of general relativity (GR). These equations combine Einstein’s GR with Maxwell’s electromagnetic theory to provide a comprehensive model for describing gravitational and electromagnetic fields in spacetime. Research into novel EMFE solutions is vital for understanding compact objects (COs) with strong gravitational fields, including white dwarfs, neutron stars, and quark stars [1,2]. The study of fluid spheres impacted by a static electric field is especially interesting. The renowned Reissner-Nordström solution [3,4] expands the exterior Schwarzschild solution. Thirukkanesh *et al*. [5] used a systematic procedure to produce new solutions for the Einstein-Maxwell equations in static, spherically symmetric spacetime, beginning with uncharged solutions. They presented a model consistent with the physical properties of a realistic charged star, ensuring continuity with the external Reissner-Nordström metric at the pressure-free boundary. This study supports similar approaches to investigating the electrification of realistic uncharged models. In these cases, gravitational attraction is balanced by both the pressure gradient and the repulsive Coulomb force [6]. The energy density of the electric field significantly contributes to the gravitational mass of an object [7]. Numerous studies have been carried out to examine the effects of electric charge on relativistic compact stellar systems [8–13]. Since Bonnor’s [14], pioneering work charged self-gravitating anisotropic fluid spheres have been the subject of extensive study in GR. The stability and equilibrium conditions of COs are improved by the presence of a static anisotropic electric field [15–17]. Neslušan investigated the global electrostatic charge of stars, proposing that asymmetries in the ionization of the primordial gas cloud or the influence of strong electromagnetic fields could lead to a net charge during a star’s formation [18]. The second scenario, as described by Omukai [19], focuses on charge accumulation through accretion, where compact stars interact with surrounding plasma and electromagnetic fields to gradually acquire charge. Furthermore, we emphasize the astrophysical significance of charged stars, noting that even a small net charge can profoundly affect their structure and stability due to the interplay of electromagnetic and gravitational forces. These insights provide a robust foundation for exploring charged stars within the context of the Einstein-Maxwell framework discussed in the article.

The anionotropy factor is an important topic to explore while studying star formation. The study of anisotropic fluid is used to simulate the pressure components of CO. In GR, anisotropic fluids were extensively studied in spherical symmetry. Bower and Liang [20] used the generalized hydrostatic equilibrium condition (TOV equation) for relativistic objects to evaluate characteristics of anisotropic fluid distribution. Cosenza *et al*. [21] developed the heuristic procedure to obtain interior solutions to Einstein’s equations by assuming anisotropic matter. Herrera *et al*. [22] investigated the behavior of self-gravitating spherically symmetric dissipative fluids with anisotropic stresses using the entire system of equations for general relativistic evolution. Herrera and Barreto [23] developed a framework for modeling Newtonian polytropes to demonstrate stellar structure. Reddy *et al*. [24] investigated the development of a spherically symmetric stellar body under anisotropic stresses and heat dissipation during gravitational collapse. They found that the temperature and core instability of the collapsing bodies increased by pressure anisotropy.

The stability analysis of CO plays a major role in mathematical modeling. The hydrostatic equilibrium equations were developed by Bondi [25] to analyze the stability of CO. Researchers have used both Schwarzschild and isotropic coordinates to develop their models. Using a cosmic Chaplygin fluid, Malaver and Kasmaei presented a unique model for compact stars with charged anisotropic matter [26]. Tello-Ortiz *et al*. [27] used modified Chaplygin (EoS) to obtain an anisotropic fluid solution for EMFEs.

Malaver *et al*. [28] found new solutions to Einstein’s field equations (EFEs) in Buchdahl spacetime, which included a nonlinear electromagnetic field. The model’s stability was examined using the radial sound speed and the Tolman-Oppenheimer-Volkoff (TOV) equations. Similarly, Maurya *et al*. [29] suggested a singularity-free model for charged compact stars under *f*(*Q*) gravity. Their findings imply the possibility of charged stars in nature and suggest that departures from classical gravitational theories may be found in future astrophysical research.

Maurya *et al*. [30] developed an exact solution aligned with a static, spherically symmetric geometry, providing valuable insight on compact star systems and the modified *f*(*Q*) gravity theory. Their proposed model was rigorously tested and met essential physical conditions for star matter. Furthermore, the study highlighted the interaction of *f*(*Q*) gravity factors, demonstrating how the coupling strength and size of the object affect its overall mass. This paper emphasizes the potential of the modified *f*(*Q*) gravity framework for future developments and practical applications. Maurya *et al*. [31] investigated compact star properties using the f(𝒯) gravity framework, focusing on neutron stars. Stability tests and observational data demonstrate that the maximum star mass exceeds $3M_{⊙}$, indicating that teleparallel gravity is acceptable for modeling huge COs. Maurya *et al*. [32] investigated an anisotropic solution for a compact star in *f*(*Q*) gravity theory with a null complexity factor. It generalizes the perfect fluid solution into an anisotropic domain with zero complexity, revealing pressure anisotropy and a constant controlling energy flow between the perfect fluid and generic fluid matter distributions. Sultana *et al*. [33] studied that the modified generalized Chaplygin gas exhibits an EoS that has been analyzed in the context of interacting scenarios. Phantom behavior is observed through reconstructed EoS parameters, indicating unique properties in modified gravity frameworks. Malaver *et al*. [34] introduced a novel model for compact stars characterized by a charged anisotropic matter distribution. This model is based on an extended form of the Chaplygin equation of state (EoS) and adheres to all the physical conditions required for a realistic stellar structure. The formulations for radial pressure, energy density, metric coefficients, anisotropy, and mass are explicitly defined and exhibit regular behavior within the star’s interior.

The analysis of CO can provide theoretical insights into dense matter systems. Advances in theoretical modeling have made it easier to translate theoretical perspectives into analytical solutions. The choice of metric potential plays an important role in finding the analytical solutions for EFEs. For evaluating the exact solutions of EFEs, Delgaty and Lake [35] established metric ansatzes for the spherically symmetric distribution of ideal fluids in static regime. These solutions are useful for estimating CO’s possible physical properties. For analyzing the characteristics of CO, the Buchdahl-I metric [37] is extremely helpful. Tamta and Fuloria [38] employ Buchdahl metric potential to investigate anisotropic stellar objects. They observed that the models retained the stability requirements across a range of parametric values. Maurya *et al*. [39] investigated anisotropic CO using the Buchdahl metric ansatz. They approximated EoS as a linear function of density.

The regime of stellar astrophysics is greatly influenced by the theory of gravity. Furthermore, theory of gravity plays a significant role at both the cosmic and galactic scales by predicting the presence of observable stable COs. In fact, the study and analysis of CO plays an essential role in astrophysics because they serve as an excellent laboratory for studying dense matter under difficult circumstances, such as strong gravity. Romani *et al*. [40] looked at the mass of pulsar PSR J0952-0607, the Milky Way’s fastest known rotating neutron star. This pulsar, whose spin period is *P* = 1.41 ms, was initially reported in [41]. It is a “black widow" pulsar, radiating and evaporating its low-mass companion due to the pulsar’s brightness. PSR J0952-0607’s mass measurement shows a maximum mass of 2.52 $M_{⊙}$ in [40]. Tangphati *et al*. [42] tests modified gravity theories using gravitational-waves data, specifically GW 190814. They predicts the existence of quark stars in the color-flavor-locked phase of color superconductivity, which aligns with observed data, imposing constraints on theoretical models. Miller *et al*. [43] studied the millisecond pulsar PSR J0030+0451, which spins at a frequency of 205.53 Hz, has been analyzed using Bayesian inference methods. This study estimate its mass $1.44_{-0.14}^{+0.15}\;M⊙$ and its radius is $R=13.02_{-1.06}^{+1.24}$ km. The millisecond pulsar J0740+6620 has a mass of $2.14_{-0.09}^{+0.1}\;M⊙$, according to the most recent accurate estimation of a CO’s maximal mass using Shapiro delay and recent studies of pulsars [44].

Maurya *et al*. [45] explored compact stars in a complexity-free background, focusing on dark matter-induced anisotropy affecting metric potentials. They discussed how dark matter in halos can increase compactness, leading to gravitational wave echoes, particularly in relation to observed compact stars. Maurya *et al*. [46] rigorously examined compact objects in *f*(*Q*) gravity, focusing on mass-radius relations and dark matter effects, particularly in the context of GW 190814 and GW 200210, while addressing stability and constraints on mass-radius measurements of supermassive compact stars. Maurya *et al*. [47] investigated gravitational decoupling in *f*(*Q*) gravity, employing a quadratic EoS to model compact objects, including the secondary component of GW 190814, revealing that this approach can constrain stellar masses and radii beyond $2.0M_{⊙}$. Maurya *et al*. [48] studied the compact star for anisotropic models. They noted that all anticipated physical properties associated with the stellar fluid distribution are present, affirming the validity of the proposed model. Maurya *et al*. [49] examined precise models of dense relativistic stars with anisotropic pressures within the framework of Buchdahl-type spacetime geometry. By reformulating the Buchdahl condition into an Euler-Cauchy equation, they derived an exact solution that satisfies all physical criteria. In another study, Maurya *et al*. [50] analyzed the influence of electric field gradients on the secondary component of the binary compact system GW 190814. Utilizing general relativistic equations, they developed and evaluated a model demonstrating smooth behavior and an outward-directed electric force. The model exhibits stability, with an increase in charge reducing pressures and central adiabatic index. It also captures the stiffness of the equation of state (EoS), and the mass derived for a slowly rotating star surpasses that of non-rotating cases. Singh *et al*. [51] conducted comparative analyses of various solutions to the field equations, including embedding class one, conformally flat, vanishing complexity factor, and conformally symmetric solutions. By employing bridge equations, they simplified the problem to a single metric potential. Their findings reveal that the class one solution adheres to a quadratic EoS, the vanishing complexity factor solution aligns with a linear EoS, the conformally flat solution accommodates both normal and exotic matter, and the conformally symmetric solution follows a cubic polynomial form.

GW190814 is a binary merger involving a black hole and a compact object, possibly another black hole or a neutron star, though its precise identity remains unclear due to limited observational evidence. Neutron stars such as PSR J0952-0607, PSR J0030+0451, and PSR J0740+6620 are examples where charge accumulation likely occurs through interactions with their environment or accretion processes. Similarly, pulsars like PSR J1614-2230 and PSR J2215+5155, along with accreting neutron stars like 4U 1608-52, appear to have gained charge over time through evolutionary and environmental factors rather than being born with it. Malaver and Iyer [52] investigated relativistic charged models with anisotropic pressure, focusing on the pulsar PSR J0952–0607. Bhar [53] examined the physical characteristics of charged compact stars, showing that the masses of four compact objects GW 190814, PSR J0952-0607, PSR J0740+6620, and PSR J1614-2230 can be attained for various values of α. Pradhan *et al*. [54] analyzed geometrically deformed charged anisotropic models within the framework of *f*(*Q*,*T*) gravity. They evaluated the physical viability of their models for neutron stars, including PSR J1810+174, PSR J1959+2048, PSR J2215+5135, and GW190814. Their results demonstrated stable mass profiles, regular behavior, and the absence of gravitational collapse, consistent with the Buchdahl–Andréasson limit. Mardan *et al*. [55] explored charged anisotropic compact stars using a core-envelope model with a polytropic core and a linear envelope, finding that physical parameters exhibited well-behaved behavior in both the core and envelope regions for compact stars 4U1608-52 and SAX J1808.4-3658.

The moment of inertia is challenging to measure accurately in neutron stars due to complexities in pulsar timing and the influence of kinematic effects, making it difficult to isolate its contribution in observational data. The moment of inertia is frequently overlooked in pulsar research that focuses on compact star models because the primary purpose is to calculate the star’s mass, radius, and stability. These values are sufficient to understand static or spherically symmetric arrangements. When dealing with rotational dynamics like spin development or pulsar glitches, the moment of inertia comes into play. If rotational effects are minor or neglected, the moment of inertia has little direct influence on solutions or observable comparisons [56].

The goal of this work is to create a stellar model that would account for the physical properties of COs, which could be made up of dark matter or dark energy. These characteristics include anisotropy, radial and transverse pressures, mass, radius, and energy density with charge. The radii of pulsars and other secondary objects connected to gravitational waves can be predicted by the model. To ensure the solution is physically acceptable, the following conditions are imposed [36]:

**Regularity at the Center:** Physical quantities such as density and pressure must remain finite and non-singular at *r* = 0.

**Positive Definiteness:** The density ρ and the pressures must remain positive throughout the stellar interior. The density and pressure decrease smoothly from the center to the boundary.

**Causality Condition:** The causality and cracking conditions must be satisfied. The criteria $0\leq v_{r}^{2}<1$ and $0\leq v_{t}^{2}<1$ should hold simultaneously within the stellar composition.

This paper outlined as follows. In Section 2, a static spherically symmetric spacetime is considered to derive the EMFEs using the Buchdahl metric for the *g*_*rr*_ component and the modified Chaplygin EoS. The metric potential ν, tangential pressure (*p*_*t*_), radial pressure (*p*_*r*_), anisotropy factor $\left(\Delta =p_{t}-p_{r}\right)$, and energy density (ρ) are all determined in this section. The constants in the ansatz are found by matching the interior solutions to the exterior geometry in Section 3. The development of the mass function is illustrated in Section 4, where the radii and mass values of several recent pulsars are presented in Tables 1 and 2. Section 5 provides a graphical depiction of the essential elements of stellar configuration, including causality and energy conditions. The stability of the model is analyzed in Section 6 using reliable techniques such as the generalized hydrostatic equilibrium condition (TOV equation), with results indicating that the model satisfies all relevant stability criteria. The article concludes with final remarks in Section 7.

**Table 1:** The values of mass function according to the constant *H* and $K=10^{-7}$

H	m	r
0.20	2.056	7.18
0.30	2.543	8.79
0.40	2.927	10.05
0.50	3.248	11.10
0.56	3.419	11.66
0.60	3.520	11.99
0.70	3.761	12.78
0.80	3.975	13.48
0.90	4.182	14.16
1.00	4.352	14.72

**Table 2:** Measured radius from our model of several recently discovered pulsars and relativistic objects for $K=10^{-7}$

COs	Measured mass	Measured radius	*H* = 0.5	*H* = 0.6	*H* = 0.7	*H* = 1
GW 190814 [42]	2.5-2.67	10.53-11.39	8.7	9.0	9.5	10
PSR J0952-0607 [58]	2.35±0.17	14.087±1.0816	7.5	8.0	8.5	8.7
PSR J0030+0451 [43]	$1.44_{-0.14}^{+0.15}$	$13.02_{-1.06}^{+1.24}$	5.0	5.7	6.0	6.3
PSR J0740+6620 [59]	2.08±0.07	13-15	6.9	7.2	7.4	7.6
GW 170817 [60]	1.4	9.67-13	4.8	5.0	5.2	5.5
PSR J1614-2230 [61]	1.97±0.04	13±2	6.5	6.7	7.0	7.2
PSR J2215+5135 [62]	$2.27_{-0.15}^{+0.17}$	–	8.5	8.7	9.0	9.3
4U 1608-52 [63]	1.74±0.14	9.3±1.0	5.7	6.0	6.3	6.5

## The EMFEs with modified Chaplygin EoS

The static spherically symmetric line element is represented as

$$ds^{2}=-e^{2\nu (r)}dt^{2}+r^{2}(d\theta^{2}+sin^{2}\theta d\phi^{2})+e^{2\lambda (r)}dr^{2}.$$ (1)

The EMFEs that link geometry and matter can be written as

$$R_{ij}-\frac{1}{2}g_{ij}R=8\pi (T_{ij}^{\left(m\right)}+T_{ij}^{\left(em\right)}).$$ (2)

The anisotropic representation of $T_{\alpha \beta }$ static matter distribution is provided as

$$T_{ij}^{\left(m\right)}=[\begin{array}{cccc} -\rho & 0 & 0 & 0\\ 0 & p_{r} & 0 & 0\\ 0 & 0 & p_{t} & 0\\ 0 & 0 & 0 & p_{t} \end{array}]$$ (3)

$$T_{\nu }^{\mu (em)}=\frac{1}{4\pi }[F_{\nu k}F^{\mu k}-\frac{1}{4}g_{\nu }^{\mu }F_{\sigma k}F^{\sigma k}].$$ (4)

The Maxwell electromagnetic field equations can be expressed as

$$(\sqrt{-g}F^{\mu \nu }{)}_{,\nu }=4\pi \sqrt{-g}J^{\mu },$$ (5)

$$F_{\left[\mu \nu ;\sigma \right]}=0,$$ (6)

where σ stands for the five-vector for electric current and electrical conductivity is shown by the expression $J^{\mu }=\sigma u^{\mu }$. Here, the symbols “," and “;" denote, respectively, covariant derivative and partial differentiation with respect to the given coordinate.

The EMFEs are obtained by applying (Eqs. 1), (3) and (4) to (Eq. 2).

$$\frac{2e^{-2\lambda }\lambda^{\prime }}{r}+\frac{1}{r^{2}}-\frac{e^{-2\lambda }}{r^{2}}=8\pi \rho +E^{2},$$ (7)

$$\frac{2e^{-2\lambda }\lambda^{\prime }}{r}+\frac{1}{r^{2}}-\frac{e^{-2\lambda }}{r^{2}}=8\pi \rho +E^{2},$$ (8)

$$e^{-2\lambda }\left(\nu^{\prime \prime }+\nu^{\prime 2}-\lambda^{\prime }\nu^{\prime }-\frac{\lambda^{\prime }}{r}+\frac{\nu^{\prime }}{r}\right)=8\pi p_{t}+E^{2},$$ (9)

and


$$\left[\begin{array}{c} r^{2}E \end{array}\right],r=4\pi r^{2}\sigma e^{(\lambda +\mu )/2}.$$ (10)

The derivative with respect to *r* is represented by (′). (Eq. 10) represent the electric field *E* as

$$E(r)=\frac{1}{r^{2}}\int_{0}^{r}4\pi r^{2}\sigma e^{\left(\lambda +\mu \right)/2}dr=\frac{q(r)}{r^{2}},$$ (11)

where *E*(*r*), representing the total electric charge within a sphere of radius *r*, which is independent of the coordinate *t*. The value of the electric charge is proposed by Prasad, *et al*., [36] as

$$E^{2}=\frac{C^{2}}{2(1+Cr^{2}{)}^{2}},$$ (12)

where *C* is any constant. The Buchdahl-I metric [37] is considered in way that described in [35] as

$$e^{2\lambda (r)}=\frac{2+2\chi r^{2}}{2-\chi r^{2}},$$ (13)

where ${\text{Km}}^{-2}$ is the dimension of the constant χ. The energy density ρ is expressed by using (Eq. 13) in (Eq. 7) as

$$\rho =\frac{3\chi^{2}r^{2}}{16\pi (\chi r^{2}-2)(\chi r^{2}+1)}-\frac{9\chi }{8\pi (\chi r^{2}-2)(\chi r^{2}+1)}-\frac{C^{2}}{16\pi (1+Cr^{2}{)}^{2}}.$$ (14)

The form of the modified Chaplygin EoS is given in [57] as

$$p_{r}=H\rho -\frac{K}{\rho^{n}},$$ (15)

where *n*, *H* and *K* are constants. Taking *n* = 1 the value of the metric potential ν can be obtained by using (Eqs. 13), (14), (15) in ([5]Eq. 8).

V=−132πr7(2−r2χ)(1+r2χ)3(32πq2r4(1+r2χ)4−32πr6(1+r2χ)4−16πr6(r2χ−2)(1+r2χ)3−16Hπr4(3r4χ(3+r2χ)+2(q+qr2χ)2)×(1+r2χ)2−K(3r4χ(3+r2χ)−2(q+qr2χ)2)2), (16)

Similarly, radial and tangential pressure can be obtained as

pr=3Hr2χ216π(χr2−2)(χr2+1)−9Hχ8π(χr2−2)(χr2+1)−C2H16π(1+Cr2)2−8Kπ3χ(r2χ−6)2(χr2+1)(χr2−2)−C22(1+Cr2)2, (17)

pt=2−r2χ16π(1+r2χ)[−2C3r(1+Cr2)3−C22(1+Cr2)2−ζr+1+r2χri(2−r2χi)(−4rχ(−2+r2χ)(2+2r2χ)2+2rχ2+2r2χ+64Kπ2r2αβ2−128Kπ2rβ+2Hrβ+Hr2α)+2χ(γ+Hr2β)2−r2χ+2χ(1+r2χ)(γ+Hr2β)(2−r2χ)2−(1+r2χ)(γ+Hr2β)r2(2−r2χ)+ηr−ζη+η2], (18)

where


α=2C3r1+Cr23−3rχ2(−6+r2χ)−2+r2χ1+r2χ2−3rχ2(−6+r2χ)−2+r2χ1+r2χ+3rχ2(−2+r2χ)(1+r2χ)β=3χ(−6+r2χ)2(−2+r2χ)(1+r2χ)−C22(1+Cr2)2γ=1−64Kπ2r2β+−2+r2χ2+2r2χ,ζ=4rχ2−r2χ+4rχ(1+r2χ)(2−r2χ)2,η=C22(1+Cr2)2+(1+r2χ)(γ+Hr2β)r(2−r2χ).

The expression for total charged gravitational mass of sphere is given as

$$m(r)=4\pi \int_{0}^{R}[\rho +\frac{E^{2}}{8\pi }]r^{2}dr.$$ (19)

## Exterior spacetime and junction conditions

The exterior Reissner-Nordström spacetime is matched to the interior spacetime at the boundary surface to determine the continuity of metric potentials at that surface. The external Reissner-Nordström metric is expressed as

$$ds^{2}=-f(r)\;dt^{2}+\frac{1}{f(r)}\;dr^{2}+r^{2}\left(d\theta^{2}+\sin^{2}\theta \;d\phi^{2}\right),$$ (20)

where the function f(r) is defined as


$$f(r)=1-\frac{2M}{R}+\frac{q^{2}}{r^{2}}.$$

The continuity of the two metric potentials at the boundary surface of the CO result as

$$e^{2\nu }=e^{-2\lambda }=1-\frac{2M}{R}+\frac{q^{2}}{r^{2}},$$ (21)

The *p*_*r*_ is defined as zero at the boundary surface, i.e.,

$$p_{r}(R)=0.$$ (22)

The expressions of constants χ and *K* can be obtained by using (Eq. 21), (Eq. 13), (14) and (15)

$$\chi \;=\;\frac{C^{2}R^{2}-8CR^{2}u-4C^{2}R^{4}u-4u}{R^{2}\left(-3-6CR^{2}-C^{2}R^{2}-3C^{2}R^{4}+8CR^{2}u+4C^{2}R^{4}u+4u\right)},$$ (23)

$$\begin{array}{rcl} K & = & \frac{H}{256\pi^{2}}{\left(\frac{C^{2}}{(1+Cr^{2}{)}^{2}}-\frac{3\chi (-6+r^{2}\chi )}{\left(-2+r^{2}\chi )(1+r^{2}\chi \right)}\right)}^{2}. \end{array}$$ (24)

Both the *p*_*r*_ and the ρ have to be positive and finite at the center. (Eq. 14) expresses the central density as

$$\rho_{0}=\frac{9\chi }{16\pi }-\frac{C^{2}}{16\pi }.$$ (25)

After substituting the value of χ given in (Eq. 23) into (Eq. 25), obtained as

$$\rho_{0}=\frac{9(C^{2}R^{2}-4u-8CR^{2}u-4C^{2}R^{4}u)}{16\pi R^{2}(-3-6CR^{2}-C^{2}R^{2}-3C^{2}R^{4}+4u+8CR^{2}u+4C^{2}R^{4}u)}-\frac{C^{2}}{16\pi }.$$ (26)

The expression for central pressure is derived from ([12] Eq. 17) as


$$p_{r}(0)=\frac{9H\chi }{16\pi }-\frac{C^{2}H}{16\pi }-\frac{16K\pi }{9\chi -C^{2}}.$$

The positivity of the ρ is only guaranteed for χ>0, according to (Eq. 25). It is observed that under this idea, given the positive central pressure, there are certain limitations on parameters *H* and *K* in this model that follow the inequality as

$$\frac{H}{K}>\frac{256\pi^{2}}{(9\chi -C^{2}{)}^{2}},$$ (27)

i.e., the radius *R* and compactness u of the star determine the ratio $\left(\frac{H}{K}\right)$. Consequently, it was not possible to choose *H* and *K*’s values at random. We have selected *H* and *K* values in accordance with (Eq. 27).

## 1 Mass function

**Fig 1:**
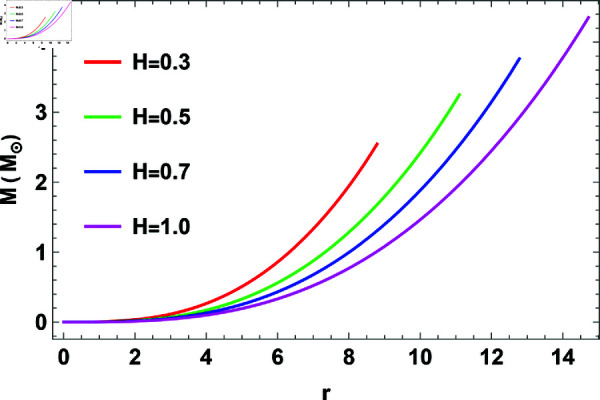
The plot of mass function against r.

Fig 1 display the mass of the model for different values of *H* and *K*. We observed a range of masses from 2.056 – 4.352 and their associated radii from 7.18–14.72 km for different values of *H*, *n* = 1, and *K* = 10^−7^. Table 1 shows that when *H* increases the mass and radius is also increase. Table 2 shows that changing the values of *H* and *K* use to predict the radii of COs. Therefore, the presented model can be used to estimate the radii of a studied COs.

## 2 Physical implementation of the model

The properties of COs will be illustrated graphically in this section. For this, GW 190814, PSR J0952-0607, PSR J0030+0451, GW 170817, PSR J1614-2230, PSR J2215+5135, 4U 1608-52, and PSR J0740+6620 COs were considered. The measured radii and masses of these COs, along with their corresponding compactness factors, are provided in Table 2.

**Fig 2:**
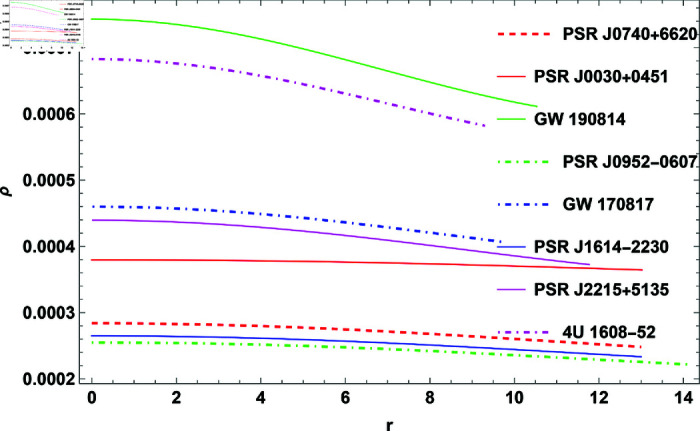
The plot of density function for H=0.3.

**Fig 3:**
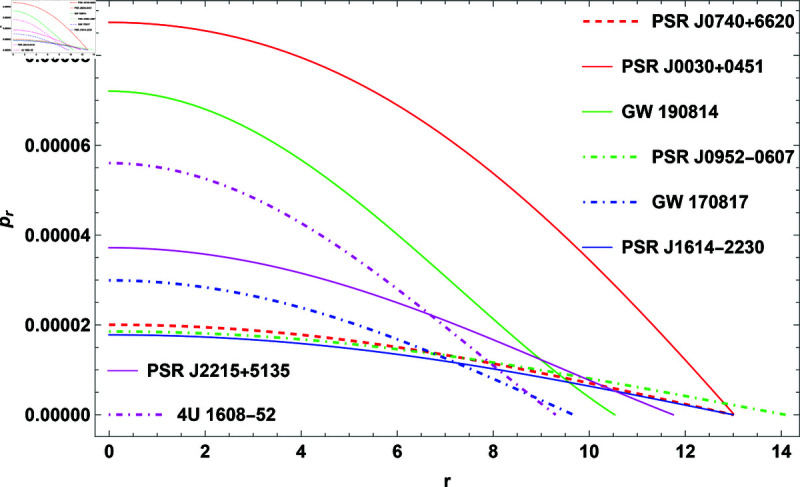
The plot of radial pressure for H=0.3.

**Fig 4:**
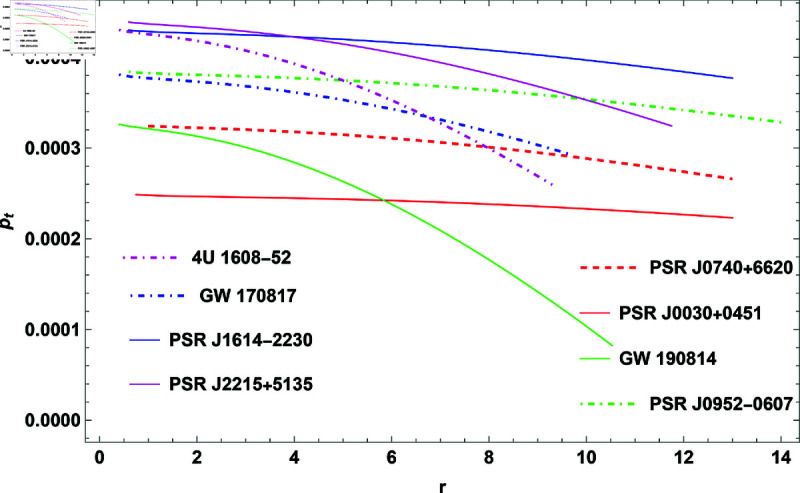
The plot of tangential pressure for H=0.3.

### Causality conditions for stability analysis

The two velocities taken into account in the causality stability analysis are the tangential $\left(v_{t}^{2}\right)$ and radial $\left(v_{r}^{2}\right)$ speeds of sound. Sound velocities’ causality conditions implies an absolute upper bound for both $v_{r}^{2}\leq 1$ and $v_{t}^{2}\leq 1$. However, the thermodynamic stability guarantees that both $v_{r}^{2}$ and $v_{t}^{2}>0$. Thus, the criteria $0\leq v_{r}^{2}<1$ and $0\leq v_{t}^{2}<1$ should hold simultaneously inside the stellar composition. Because of the difficulty of the sound velocity equations, we have graphically depicted variations of $v_{r}^{2}$ and $v_{t}^{2}$ in Figs 6a and [fig:subfig10]6b, respectively. Figs 6a and reffig:subfig10 make it clear that this model complies with the causality conditions.

### Energy conditions

The next investigation will determine whether the energy conditions are satisfied by the theoretical models [64–66]. These conditions represent essential significant events in GR, determining the allowed energy and pressure distributions throughout spacetime. The necessary conditions consist of the null, weak, strong, and dominant energy conditions. Compliance with these conditions supports the models physical validity by proving their compatibility with fundamental gravity and energy concepts. This evaluation is critical for establishing the theoretical validity and observational relevance of the findings in context of astrophysical and cosmological research. The energy conditions for current stellar configuration are fully satisfied. Energy conditions must be satisfied both within and on the surface of a compact star for a model to be considered physically realistic [67]-[68]. The energy conditions are

- Null energy condition: $\rho +p_{r}+\frac{E^{2}}{4\pi }\geq 0$, $\rho +p_{t}+\frac{E^{2}}{4\pi }\geq 0.$
- Weak energy condition : $\rho +\frac{E^{2}}{8\pi }\geq 0$
- Strong energy condition: $\rho +p_{r}+2p_{t}+\frac{E^{2}}{4\pi }\geq 0$.
- Dominant energy condition: $\rho +\frac{E^{2}}{8\pi }\geq 0$, $\rho -p_{r}+\frac{E^{2}}{8\pi }\geq 0$, $\rho -p_{t}+\frac{E^{2}}{8\pi }\geq 0$.

### 2.1 Gravitational redshift

Gravitational redshift is the process where light or electromagnetic radiation emitted from a source in a strong gravitational field shifts to longer wavelengths as it escapes the field. This shift causes the light to move toward the red end of the spectrum. GR predicts this effect, which occurs because gravity affects the energy of the escaping radiation. The degree of gravitational redshift *Z*_*s*_ is given as

$$Z_{s}=\frac{1}{\sqrt{1-\frac{2M}{r}+\frac{q^{2}}{r^{2}}}}-1$$ (28)

Bohmer and Harko [69] demonstrated that for an anisotropic star, the surface redshift could reach a maximum of $Z_{s}\leq 5$. Ivanov [71] further refined this maximum value, establishing it at *Z*_*s*_ = 5.211. Within this framework, the surface redshift for COs such as GW 190814, PSR J0952-0607, PSR J0030+0451, PSR J0740+6620, GW 170817, PSR J1614-2230, PSR J2215+5135, and 4U 1608-52 is constrained to *Z*_*s*_<1. This phenomenon becomes more pronounced near very massive objects like black holes or neutron stars, where the gravitational field is strong enough to noticeably stretch the wavelength of light. As light travels outward from such regions, its energy decreases, making it appear redder to observers in weaker gravitational fields. Furthermore, this redshift increases towards the object’s boundary.

### Adiabatic index

The adiabatic index parameter is an important mathematical tool to determine how pressure changes with density. In scenarios such as the study of neutron stars or the dynamics of gravitational collapse, the adiabatic index helps assess how “stiff" the fluid is, affecting its stability against gravitational forces. According to Newtonian approximations, a stable compact structure requires an adiabatic index greater than Γ>4/3 within the star interior. However, this criterion shifts for relativistic compact star models. Arias *et al*. [70] investigated the stability of the solution through the application of the adiabatic index criterion. The radial adiabatic index, $\Gamma_{r}$, and the tangential adiabatic index, $\Gamma_{t}$, are commonly defined as

$$\Gamma =(\frac{p_{r}+\rho }{p_{r}})\frac{dp_{r}}{d\rho },$$ (29)

$$\Gamma_{t}=(\frac{p_{t}+\rho }{p_{t}})\frac{dp_{t}}{d\rho }.$$ (30)

## Stability analysis

The investigation of TOV equation is necessary for evaluating the stability of the model under different forces. The stability of an anisotropic CO is influenced by several forces, including the hydrostatic force (*F*_*h*_), the gravitational force (*F*_*g*_), and the anisotropic force (*F*_*a*_). Under the combined effect of these factors, the model supposed to be in equilibrium. The generalized hydrostatic equilibrium condition [72]-[73] of the following form has been used to study the stability

$$-\frac{M_{G}(r)e^{\lambda -\nu }}{r^{2}}(\rho +p_{r})-\frac{dp_{r}}{dr}+\frac{2\Delta }{r}+\frac{q}{4\pi r^{4}}\frac{dq}{dr}=0,$$ (31)

where *M*_*G*_ denotes the active gravitational mass, which may be found using the Tolman-Whittaker [74] mass formula, which is as follows:

$$M_{G}(r)=r^{2}\nu^{\prime }e^{\nu -\lambda }.$$ (32)

(Eq. 32) is substituted into (Eq. 31), yields

$$-\nu^{\prime }(\rho +p_{r})-\frac{dp_{r}}{dr}+\frac{2\Delta }{r}+\frac{q}{4\pi r^{4}}\frac{dq}{dr}=0.$$ (33)

The forces represented as


$$F_{g}=-\nu^{\prime }(\rho +p_{r}),$$ (34)

$$F_{h}=-\frac{dp_{r}}{dr},$$ (35)

$$F_{a}=\frac{2\Delta }{r},$$ (36)

$$F_{e}=\frac{q}{4\pi r^{4}}\frac{dq}{dr}.$$ (37)

The expressions of (Eqs. 34)([fe]37) can be computed using (Eqs. 14), ([12]17), and (18). A graphical representation of the model’s equilibrium conditions is presented in Figs 11a–11h

**Fig 5:**
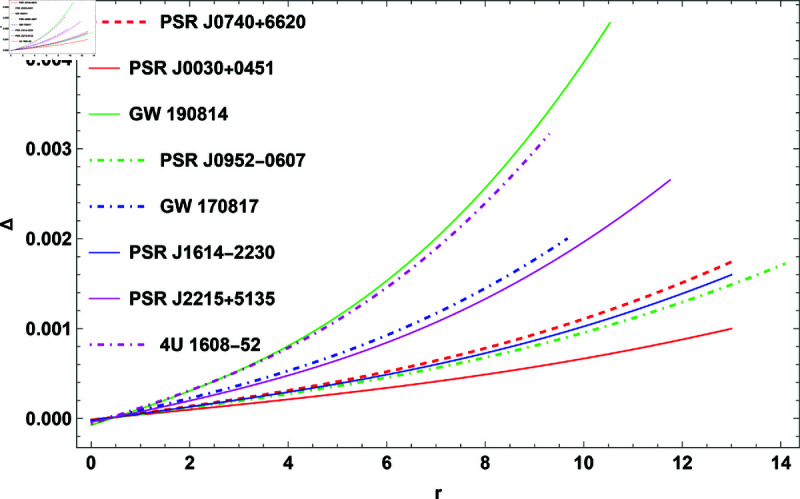
The plot of anisotropy for H=0.3.

**Fig 6:**
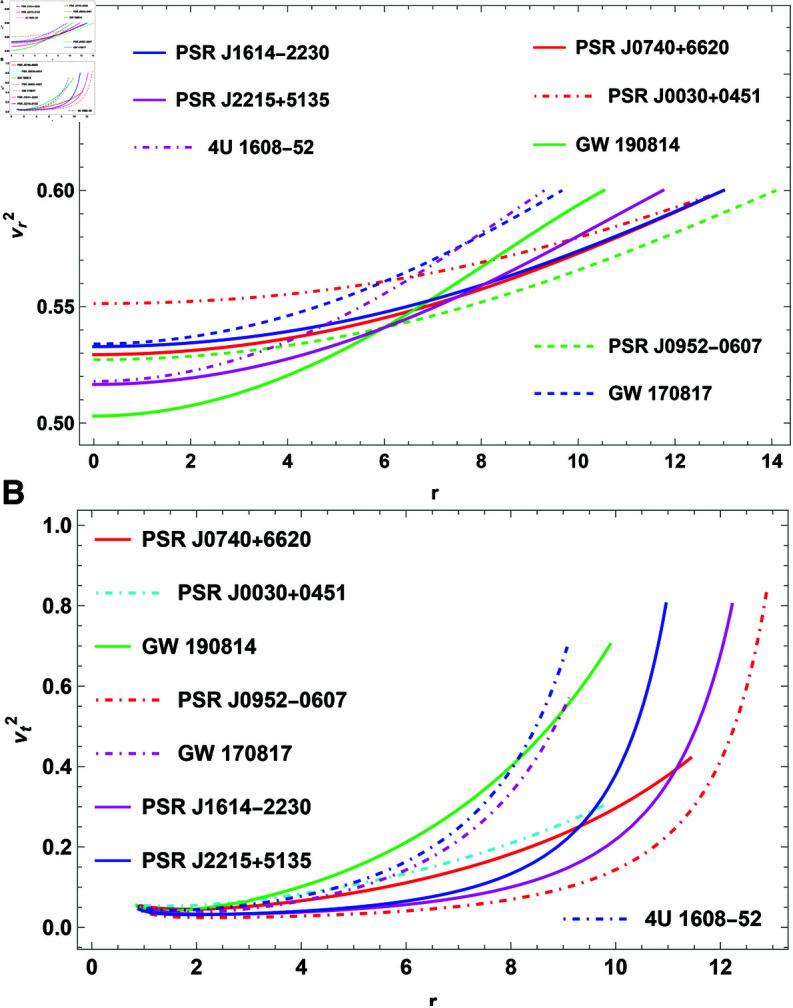
The plot of causality conditions.

**Fig 7:**
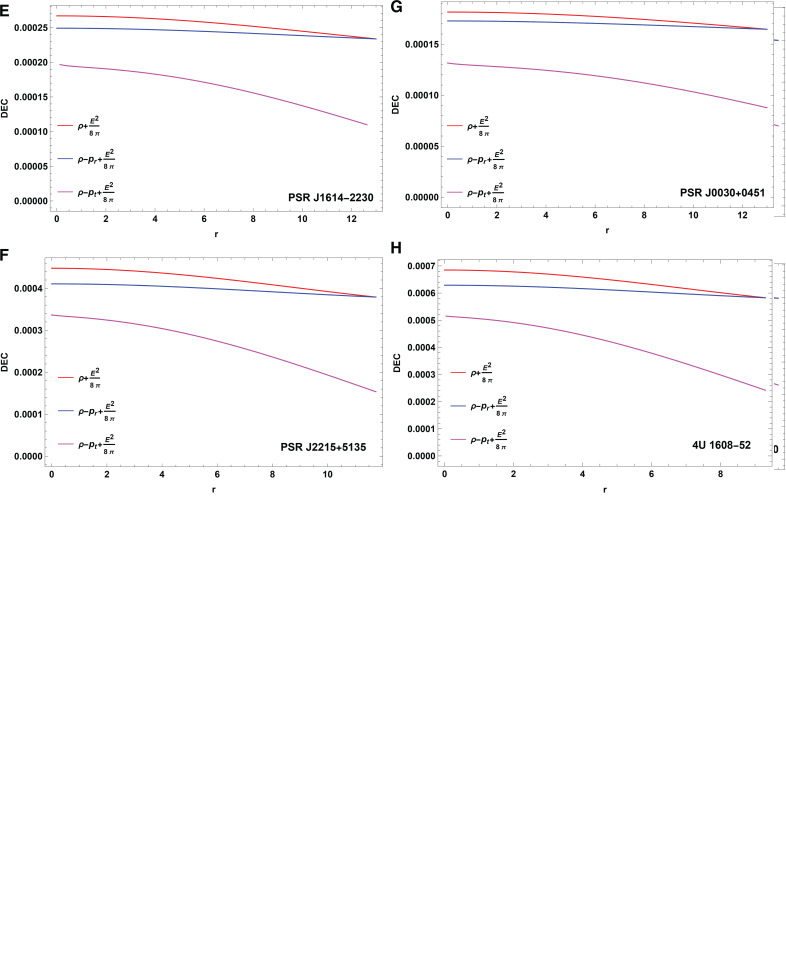
The plot of dominant energy condition against r.

**Fig 8:**
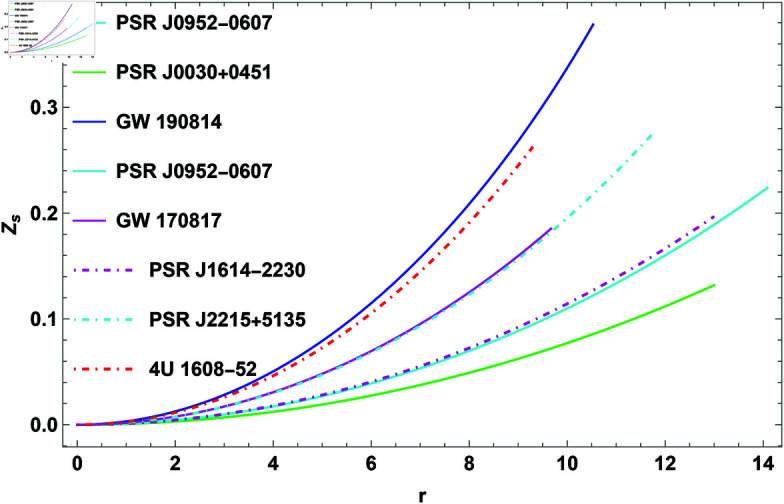
The plot of redshift for H=0.3.

**Fig 9:**
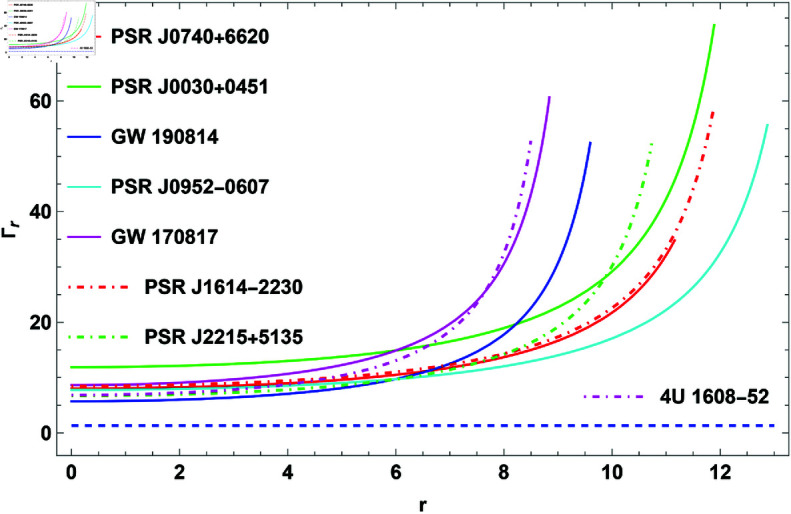
The plot of radial adiabatic index for H=0.3.

**Fig 10:**
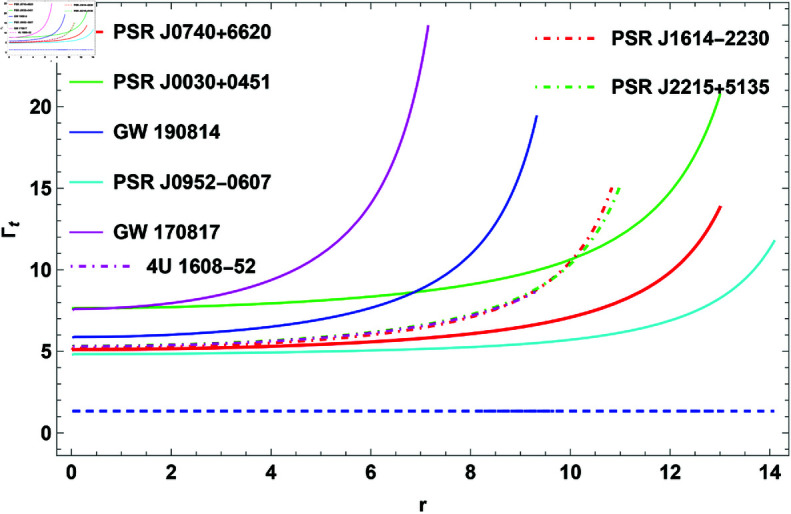
The plot of tangential adiabatic index for H=0.3.

**Fig 11:**
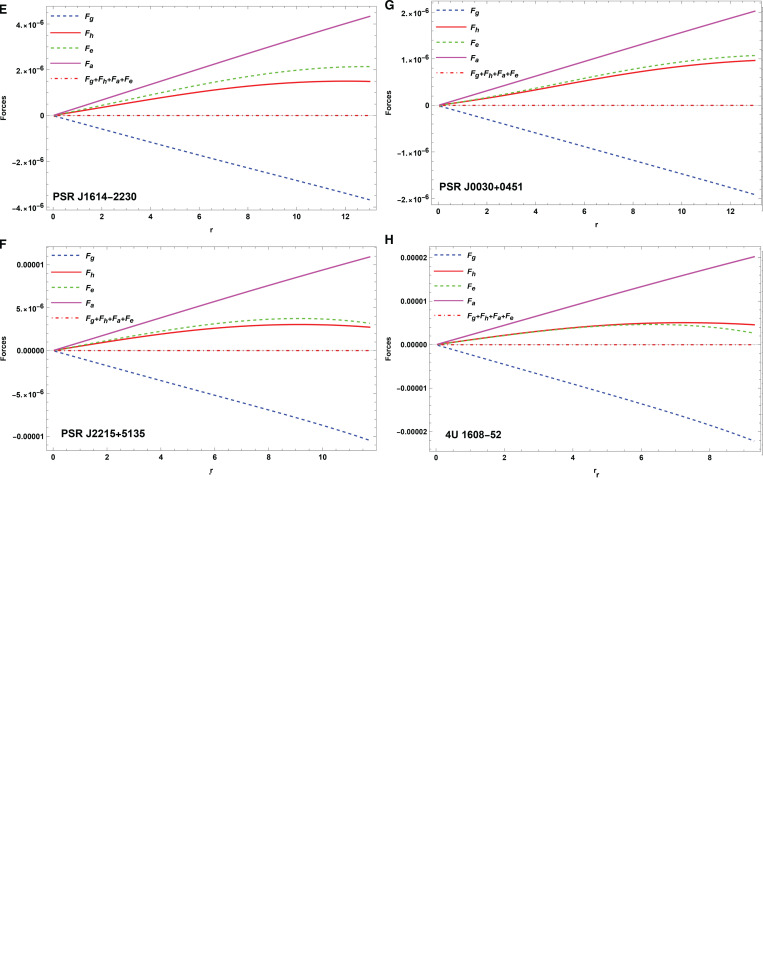
The plot of hydrostatic equilibrium for various objects

## Conclusion

This article introduces a comprehensive generalized model of charged anisotropic COs using a Chaplygin EoS and a modified Buchdahl-I metric. The main focus is on modeling the internal structure of COs, providing insight into how these objects behave under various physical conditions, such as mass, pressure, density, and anisotropy. Additionally, the study examines the equilibrium of COs under gravitational, hydrostatic, and anisotropic forces, ultimately contributing to a deeper understanding of dense stellar objects like neutron stars and pulsars. Furthermore, the analysis of stability through the causality conditions, energy conditions, gravitational redshift and adiabatic index strengthens the accuracy of this model.

**Metric continuity and constants:** The continuity of the metric functions at the boundaries of stellar objects leads to the introduction of specific constants that play a critical role in determining the properties of these objects. Specifically, the constant χ arises from the requirement of smooth transitions in metric functions across the stellar boundary, as expressed in Eq. (23). Similarly, the constant *K* in ([K]Eq. 24) ensures the proper matching of the interior and exterior solutions at the boundary surface.

The key aspect of this model is that the central density is constrained to positive values, indicated by χ>0. The central pressure, another critical factor in the equilibrium of COs, with constants *H* and *K*, as evidenced by (Eq. 27). This correlation is crucial for maintaining a stable model of stellar objects, ensuring consistency between theoretical predictions and observable astrophysical phenomena.

**Mass and radius of COs:** The mass function for different values of *H* is depicted in Fig 1, and the variation in mass with respect to the modified Buchdahl-I metric parameter *H* is detailed in Table 1. For instance, at *K* = 10^−7^, the mass varies from 2.056–4.352 solar masses, while the radius is from 7.18–14.72 km as *H* increases from 0.2–1.00. This relationship demonstrates that as the parameter *H* increases, so do the mass and radius.

The results provide an excellent opportunity to compare the mass range of lighter COs and pulsars to current observational data. For example, this model includes pulsars such as PSR J0952-0607, GW 190814, PSR J0740+6620, PSR J0030+0451, and PSR J1614-2230. Table 2 shows how varying constants predict the radius for different mass ranges. This model’s adaptability is especially useful for researching high-mass pulsars, as it shows that these objects may have a Chaplygin EoS in their interior, which is frequently linked with dense star formations.

**Density, radial pressure, tangential pressure, and anisotropy:** The EMFEs are fundamental equations in GR that describe how matter and energy influence the curvature of spacetime. By solving these equations, one can derive important physical quantities for a CO, including:

- **ρ**: Represents the mass-energy density of the object, indicating how much mass is contained within a given volume.
- ***p***_***r***_: The pressure experienced by the object in the radial direction, which affects how the object resists gravitational collapse.
- ***p***_***t***_: The pressure acting perpendicular to the radial direction, which can differ from the radial pressure in anisotropic models.
- **Δ**: A measure of the difference between the *p*_*r*_ and *p*_*t*_, which quantifies the degree of anisotropy in the pressure distribution.

(Eqs. 14), ([12]17), and (18) provide mathematical expressions for these quantities, illustrating how they depend on the radial coordinate and other parameters. The analysis shows that ρ, *p*_*r*_, and *p*_*t*_ all exhibit positive values, which is necessary for the physical stability of the model. The quantities reach their maximum values at the center of the CO, particularly when the parameter *H* = 0.3 is applied. Moving outward from the center toward the surface, these values gradually decrease. This trend is characteristic of stable stellar configurations, where central conditions are more extreme compared to the outer layers. Anisotropy significantly influences the stability of the CO. It reflects the difference between *p*_*r*_ and *p*_*t*_, with Fig 5 showing that the anisotropic pressure is zero at the center. This indicates balanced pressures at the core, ensuring a stable configuration. As one moves outward, anisotropy increases, causing *p*_*t*_ to become smaller than *p*_*r*_, which generates a repulsive force. This force opposes gravitational attraction, playing a crucial role in maintaining the stability of the object and preventing gravitational collapse. Thus, the interaction between pressures and the anisotropic force is essential for understanding the equilibrium and stability of COs within the framework of GR.

**Causality and energy conditions:** The causality condition is satisfied by the radial and tangential sound speeds, ensuring that the speed of sound within the CO does not surpass the speed of light, as shown in Figs 6a and [fig:subfig10]6b. The results show that the stellar model is physically feasible at *H* = 0.3 since the causality condition is not violated. In addition to causality, the anisotropic fluid distribution in this model satisfies several energy conditions, including the null, weak, strong, and dominant. These energy conditions are crucial for ensuring that the CO model represents a physically meaningful and stable object. The energy conditions are investigated for a range of pulsars, including those mentioned earlier, and the results, depicted in Figs 7a7h, show that these conditions reach their maximum at the core and gradually decrease toward the surface.

**Gravitational redshift:** The gravitational redshift, *Z*_*s*_, is another important characteristic of COs, representing the extent to which light is shifted to longer wavelengths due to the intense gravitational field. The redshift depends on the compactness of the CO, which is defined by its mass-to-radius ratio. The CO’s redshift is zero in its center and reaches its highest value near its surface, as depicted in Fig 8. The redshift values observed for neutron stars, which are impacted by their high gravitational fields, typically range between 0.1 and 0.4, depending on the star’s mass and radius.

**Adiabatic index** The stability of COs under radial perturbations can be measured using the adiabatic index, Γ, which quantifies the connection between pressure and density changes. For a stellar model to be stable, Γ must be greater than $\frac{4}{3}$. Fig 9 shows the behavior of Γ, indicating that the criterion $\Gamma >\frac{4}{3}$ is met for different CO configurations. These findings corroborate the stellar model’s stability in the face of radial perturbations, proving its dependability as a representation of CO.

**Hydrostatic equilibrium conditions:** The TOV equation describes hydrostatic equilibrium, which assures that COs remain balanced under gravitational, hydrostatic, and anisotropic forces. Equation (31) establishes the equilibrium, as shown in Figs 11a–11h, showing the stability of the model. This equilibrium is critical for maintaining the structural integrity of the CO.

In conclusion, the generalised modified Buchdahl-I metric, along with a modified Chaplygin EoS, provides a thorough framework for modeling charged anisotropic COs. The model accurately predicts the mass and radius of COs, including neutron stars and pulsars, while keeping to fundamental conditions such as, causality, and hydrostatic equilibrium. Stability investigation confirms its reliability, making it an important tool for researching the properties of ultra-dense COs.
